# Do women with Premenstrual Dysphonic Disorder experience differences in emotion recognition during the menstrual cycle?

**DOI:** 10.1192/j.eurpsy.2022.2218

**Published:** 2022-09-01

**Authors:** A. Vardiampasis, C. Gramandani

**Affiliations:** 1 GENERAL HOSPITAL OF RETHYMNO, Mental Health Center Of Rethymno, RETHYMNO, Greece; 2 GENERAL HOSPITAL OF RETHYMNO, Mental Health Center Of Rethymno, ΡΕΘΥΜΝΟ, Greece

**Keywords:** ERT, PMDD

## Abstract

**Introduction:**

The relationship between behavioral changes and the menstrual cycle in women at a reproductive age has been investigated in several studies; women during every menstrual cycle experience noticeable changes in levels of sex hormones which are consequently reflected on their mood and behavior. The relationship between the menstrual cycle and the emotion recognition processing has been also studied.

**Objectives:**

The aim of this study was to examine if differences exist between women with Premenstrual Dysphonic Disorder (PMDD) and women without PMDD in Emotion recognition processing across menstrual cycle.

**Methods:**

We examined 26 women with a PMDD and 30 women without PMDD, who have both visited the Mental Health Centre (aged 18-35 y.o., right handed, educational level >9 y., regular cycle duration). Women were clinically interviewed (DSM-V); also the Emotion Recognition Task (ERT) was administered in the luteal and the follicular phase.

**Results:**

Women with PMDD showed significant differences in emotion recognition depending on the the luteal and the follicular phase (according to estradiol and progesterone level) whereas women without PMDD did not present significantly different responses to the emotional stimuli.

**Conclusions:**

Our findings suggest that there is an effect of PMDD on emotional facial recognition across the two phases of the menstrual cycle. Thus, the importance of incorporating ERT in the formal clinical examination of PMDD is highlighted.

**Disclosure:**

No significant relationships.

